# Traffic-State-Dependent Cascading-Failure Dynamics in Urban Bus Networks: An Improved Coupled Map Lattice Framework

**DOI:** 10.3390/e28101115

**Published:** 2026-10-13

**Authors:** Jie Zhang, Xiaojuan Xie, Shungen Xiao, Ziqi Wang

**Affiliations:** 1 College of Information Engineering, Ningde Normal University, Ningde 352100, China; 2 College of Civil Engineering, Fuzhou University, Fuzhou 350116, China; 3 School of Mechanical Engineering, Tongling University, Tongling City 244061, China

**Keywords:** urban bus networks, cascading failures, Coupled Map Lattice, complex networks, nonlinear dynamics, traffic conditions, network vulnerability

## Abstract

Urban bus networks are exposed to cascading disruptions whose propagation depends not only on network topology but also on temporally varying traffic states and passenger-flow distributions. Existing vulnerability analyses often rely on static topology or hypothetical flows, limiting their ability to characterize traffic-dependent failure dynamics. This study develops a data-driven vulnerability framework for urban bus networks by integrating a coupled road–bus network, user-equilibrium assignment and a weighted Coupled Map Lattice (CML) model. Smart-card data are used to estimate passenger origin–destination demand, while road traffic states at four representative periods are mapped onto bus links to characterize travel-speed-dependent capacity reductions. The resulting weighted networks are evaluated using degree, strength, conventional betweenness, and traffic-flow betweenness. The improved CML model jointly incorporates topological connectivity, passenger-flow characteristics, external perturbations, and flow redistribution to simulate cascading failures under targeted and random attacks. The Harbin trunk bus network is used as an empirical case study. Results show that cascading-failure patterns are strongly traffic-state dependent: under more congested conditions, failures reach their peak propagation rate earlier. Across the tested parameter settings, perturbation intensity and coupling strength influence the timing and extent of cascade propagation, with responses varying by attack strategy and traffic condition. These findings demonstrate that incorporating time-varying traffic states and actual passenger flows can reveal vulnerability patterns that static topology-based analyses may overlook, providing a basis for time-sensitive disruption management and resilience-oriented bus operations.

## 1. Introduction

A well-functioning and efficient public transport network is essential for realizing the concept of Mobility as a Service (MaaS) and promoting sustainable transportation. With rapid socio-economic development and urbanization, the demand for mobility is increasing significantly, leading to escalating traffic congestion. In the context of Transit-Oriented Development (TOD) [1], ensuring the safety and efficiency of bus networks has become increasingly critical. Given the severe impact that service disruptions can have on both residents and the economy, assessing the vulnerability of urban public transport systems is of growing importance [2].

In recent years, researchers have increasingly focused on modeling and analyzing the vulnerability of public transport networks (PTNs). From a network operations perspective, evaluating the effects of disruptions—caused by random or deliberate attacks, equipment failures, or congestion—on PTNs is vital. Such analyses enable the design of contingency plans and scheduling strategies in response to cascading failures across multimodal transport networks. By optimizing the operational schedules of bus routes in real-time, considering the traffic dynamics and origin–destination (OD) demand at bus stops [3], it is possible to enhance the efficiency of passenger travel, alleviate crowding, and prevent resource wastage. Additionally, allocating appropriate defensive resources within the network [4,5] can help mitigate disruptions, improve system resilience, and ensure smoother transport operations.

Research on complex networks has traditionally focused on the performance of individual networks, particularly in areas such as robustness [6], vulnerability [7], and resilience [8]. However, the growing interconnections within modern society have increased the coupling and interaction between various networks. The increasing complexity of socio-economic activities has intensified the interdependence between these networks [9]. For example, metro systems rely on the electricity grid [10], and a grid failure would result in the suspension of metro services, causing congestion among passengers and potentially affecting other urban transportation systems. However, a failure in the metro system typically does not impact the electrical grid. Public transportation networks play a crucial role in meeting the daily travel needs of urban residents and alleviating traffic congestion. When sections of the road network (RN) experience congestion or closures, the bus network, which relies on the road infrastructure, also suffers, leading to a reduction in its overall capacity. As congestion spreads, it can affect the entire urban bus network (UBN) [11].

This study addresses these challenges by constructing interdependent road–bus networks. Specifically, it examines the vulnerability of the bus network to cascading failures, focusing on the impact of road congestion. Using IC bus swipe data, the study evaluates how road congestion affects bus travel speeds and passenger capacity at various times. The analysis is designed to understand the cascading effects of road disruptions on bus operations, revealing how these failures impact both travel times and carrying capacity.

Cascading failures in urban transit networks often arise from road disruptions or congestion, leading to significant changes in the network topology. These failures increase travel costs for passengers and degrade the overall performance of the transportation network. As a result, researchers have begun to explore the dynamics of cascading failures, focusing on passenger flow through stations and links. The scale and propagation of failures can vary depending on different transit modes. Related studies have investigated cascading failures in other complex networks. Liu et al. [12] examined cascading failures in scale-free wireless sensor networks, identified a critical load associated with large-scale failures, and proposed a corresponding control method. Li et al. [13] investigated cascading crashes induced by individual heterogeneity in complex networks. These studies provide a broader context for examining cascading failures in urban bus networks, where passenger flows and road traffic conditions must also be considered. In this paper, we propose an improved Coupled Map Lattice model to analyze the vulnerability of public transportation networks, providing insights into how cascading failures impact urban transit systems.

The remainder of this paper is organized as follows. A comprehensive literature review is presented in Section 2. The model of network construction is proposed in Section 3. The vulnerability assessment model based on CML is described in Section 4. Section 5 uses the Harbin trunk bus as an example for the vulnerability analysis. Finally, discussions are presented in Section 6.

## 2. Literature Review

### 2.1. The Research of Vulnerabilities Analysis of Bus Network

Extensive research has been conducted on the vulnerability of public transportation networks (PTNs) from various perspectives, though most studies have focused on individual systems, such as metro networks [14] or bus systems [15]. For example, Wu et al. [16] analyzed the topological characteristics of the Beijing public transport system and evaluated its vulnerability. De Los Santos et al. [17] employed a robustness index to measure the vulnerability of bus systems. Geographic Information Systems (GIS) offer real-time transportation data, providing valuable support for vulnerability assessments of urban transportation systems [18]. However, this approach heavily relies on a large volume of accurate data and is primarily limited to performing what-if scenario analyses. Hong et al. [19] introduced the concept of complementary sites, defining geographically close locations as complementary and examining the relationships between transportation subsystems. To assess system vulnerability under disruptions, it is essential first to establish a metric for system functionality. Many studies use topological metrics to evaluate transit system functionality, such as shortest path length [20], network efficiency [21], and maximum connected subgraphs [22]. However, researchers have recognized that with societal development and the optimization of transportation networks, purely topological measurements are no longer sufficient to assess network vulnerability [23]. Consequently, more recent research has begun to utilize metrics based on network accessibility [24] and passenger flow [25]. Barnhart et al. [26] examined the vulnerability of the aviation system by considering passenger delays, while Cats [27] quantified the vulnerability of PTNs by measuring the number of disrupted and delayed passengers and the severity of those delays. Lu et al. [28] proposed an improved Coupled Map Lattice (CML) model to analyze passenger travel time losses resulting from cascading failures. Zhang et al. [29] further extended this approach by integrating the CML model with a passenger flow redistribution rule to study cascading failures in urban rail transit networks. Public transportation networks are vital to urban life, providing essential services such as commuting, healthcare, commerce, and education [30]. Zhang et al. [31] compared preventive and emergency control strategies for cascading failures in large-scale bus transit networks and proposed an emergency route-relinking approach that exploits internal network redundancy. The condition of road infrastructure is directly related to the safety, orderliness, and efficiency of PTNs. Consequently, real-time road conditions have a significant impact on PTN operations. Despite this, few studies have specifically examined how road congestion affects the performance of bus networks, and even fewer have addressed the dynamic functionality of bus systems in this context.

In addition to system functionality indicators, disruptive events play a critical role in vulnerability analysis. Various disruptions have been studied in relation to public transportation systems, including earthquakes [32], floods [33], environmental degradation [34], and interference in coverage areas. This paper focuses on the interaction between road networks (RNs) and urban bus networks (UBNs), analyzing the vulnerability and capacity changes in bus networks under road congestion conditions.

### 2.2. The Applications of Interdependence Network Theory in Transportation Field

The study of interdependent networks, particularly in the context of urban bus networks (UBNs), began relatively late. Baldyrev et al. [35] analyzed the bidirectional dependencies between the Italian power grid and the internet nearly a decade after the importance of interdependent network analyses was first highlighted in relation to cascading network failures. From a network science perspective, topological methods examine the interdependencies of networks, considering both their structural and physical connections. Marashi K et al. [36] utilized correlation metrics and a heuristic causality analysis to identify the interdependencies within a cyber-physical system. In cases where a network node or link fails, many researchers analyze interdependent networks based on topological metrics. Duenas-Osorio et al. [37] proposed a methodology that uses topological indicators—such as node degree, clustering coefficient, average path length, and connectivity to measure network interdependency. This approach combines failure probability with the resulting consequences to assess network robustness. Franchina et al. [38] introduced a time-dependent cascading-failure model to investigate how interdependencies between critical infrastructure networks evolve. Belle et al. [39] developed a model for analyzing the vulnerability of interdependent railway and power networks, accounting for realistic coupling through traction power systems. However, this model only uses collected data to outline potential interdependencies and does not quantify how functional factors influence these interdependencies under varying failure scenarios. Zhang et al. [40] divided the railway network into multi-layered networks according to different train numbers, demonstrating that the railway system exhibits community structures where high-degree nodes play crucial roles. Similarly, Aletta et al. [41] argue that most studies on urban public transport overlook the system’s multi-level nature. They addressed this gap by constructing a multi-layer public bus network (PBN), where each layer represents a bus route, and evaluated how the multi-layer structure affects passenger travel. Yildirimoglu and Kim [42] identified pockets of activity and community structures within urban networks, further supporting the value of multi-layered approaches.

Recent research has increasingly focused on the vulnerability of interdependent transport networks, particularly in terms of cascading-failures. Ma et al. [43] developed a Nonlinear Load-Capacity Model to investigate cascading failures in the urban bus–subway double-layer network. Shen et al. [44] introduced a cascade failure model that incorporates mutual traffic redistribution while accounting for the traffic loss commonly observed during real-world network disruptions. They applied this model to evaluate the vulnerability of the Nanjing bus–metro network. Liu et al. [45] designed a cascading failure model using real-world data from the Chengdu–Chongqing urban agglomeration, analyzing failures in both syncretic and individual transport networks. Yin et al. [46] further examined the unique characteristics of different bus routes, including their dependencies and the heterogeneity of transfer channels. In this study, we abstract each bus line as a distinct layer in a multi-layer network. Bus stations are represented as nodes, and the distances between adjacent stations are modeled as edges within each single-layer network. This multi-layer network structure allows for a more nuanced analysis of vulnerability and cascading failures within urban bus systems.

Recent studies have further examined disruption management and passenger-flow dynamics in interdependent public transport networks. Du et al. [47] investigated resilience optimization in a bus–metro double-layer network under extreme weather events. Guo et al. [48] modeled cascading-failure and recovery propagation under time-varying passenger flows, while Hu et al. [49] incorporated temporal flow variations and metro travel preferences into robustness analysis. These studies highlight the importance of functional interactions, passenger redistribution, and temporal variability. The present study focuses on how different road traffic scenarios enter a weighted CML framework for bus-network cascading-failure analysis.

In summary, some traffic flow models focus on virtual traffic flows based on network topology features or hypothetical traffic flows in analyzing traffic networks. However, the real data used in the study of dynamical flow models are few. Interaction influences between networks are ignored in the construction of interdependent networks. In addition, some studies usually focus only on structural or functional vulnerability and fail to analyze network vulnerability from multiple perspectives. Therefore, the multi-time segment flow of UBNs is obtained by the actual road travel speeds at different moments and the user equilibrium model. Moreover, the model of the improved CML and vulnerability analysis are proposed considering a public transport network weighted by actual passenger flows of different moments.

## 3. Method of Public Transport Network Based on Complex Network

In this section, the complex network theory is used to introduce the basic network characteristics of UBNs. The stations and links of the UBNs are described by the nodes and edges of a graph, can be represented by a graph $G=\left(V,E\right)$, and $V=\{V_{i}|i=1,2,\ldots ,N\}$ is the set of nodes, $E=\{E_{ij}=\left(i,\;j\right)|i,j=1,2,\ldots ,N\}$ is the set edges of the graph, and G is the network of UBNs; the N is the total nodes of the G. Then, the origin–destination (OD) of bus network is calculated by the number of stations traveled by passengers and individual travel characteristics. Then the road network is given which weights with the current traffic flow in UBNs based on real-time traffic dynamics and analyzes the network vulnerability of urban road networks under deliberate attacks and random attacks.

### 3.1. The Indicators of Crucial Node

This section introduces four indicators for evaluating the importance of network nodes based on complex network theory and traffic flow theory. The indicator of node degree represents the number of edges connected to the node and is defined as follows:

(1) $$d_{i}=\sum_{i\neq j,j\in N}^{N}e_{ij}$$

where $d_{i}$ represents the degree of the node $v_{i}$, j is the set of nodes connected to $v_{i}$. It can be seen from the following that the larger the node degree is, the larger the number of edges connected to it, so $d_{i}$ can reflect the local connectivity of the node.

The node strength indicates the sum of the flows of the edges connected to the given node, as follows:

(2) $$S_{i}=\sum_{j=1,j\neq i}^{n}f_{ij}$$

where $S_{i}$ is the strength of the nod $v_{i}$, j are the neighborhood nodes of the $v_{i}$, and the $f_{ij}$ passage flow of the edge $e_{ij}$.

In addition, the betweenness ($B_{i}$) of node $v_{i}$ is described by the number of the shortest paths between all pairs of nodes through node $v_{i}$ and it is given as follows:

(3) $$B_{i}=\frac{\sum_{j=1}^{N}\sum_{k=1}^{N}\sigma_{jk}^{i}}{\sum_{j=1}^{N}\sum_{k=1}^{N}\sigma_{jk}}$$

where $\sigma_{jk}$ is the number of the shortest paths of nodes $v_{j}$ to $v_{k}$, $\sigma_{jk}(v_{i})$ is the number of the shortest paths of nodes $v_{j}$ to $v_{k}$ through the node $v_{i}$. The betweenness is used to indicate the road capacity, so intersections with high betweenness are important for the road capacity.

The edges relationship of the road section is considered as the calculation of the shortest path in the description of the node betweenness. However, traffic flow is an important attribute of measuring the performance of the actual road network; an improved indicator, namely traffic flow betweenness centrality (*TFB*), which considers passenger flow, was introduced as follows:

(4) $$TFB_{i}=\frac{\sum_{j=1}^{N}\sum_{k=1}^{N}F_{jk}(v_{i})}{\sum_{j=1}^{N}\sum_{k=1}^{N}F_{jk}}$$

where $F_{jk}$ denotes the total number of the traffic flow from $v_{j}$ to $v_{k}$; $F_{jk}(v_{i})$ denotes the number of passengers through vi of $F_{jk}$. If the disruption occurs at a node, then all vehicles through that node must be detoured, which will result in an increased trip distance and travel time.

### 3.2. Interdependence Network Construction of Road–Bus Network

The traffic situation refers to the road environment and vehicle operation status and trends in the traffic network—the current state and development trend of the vehicle operating environment at a certain moment or period of a certain range of road traffic networks [50]. Apps such as Google Maps, AMAP and other apps are used by people in daily travel to plan their travel routes.

**Figure 1:**
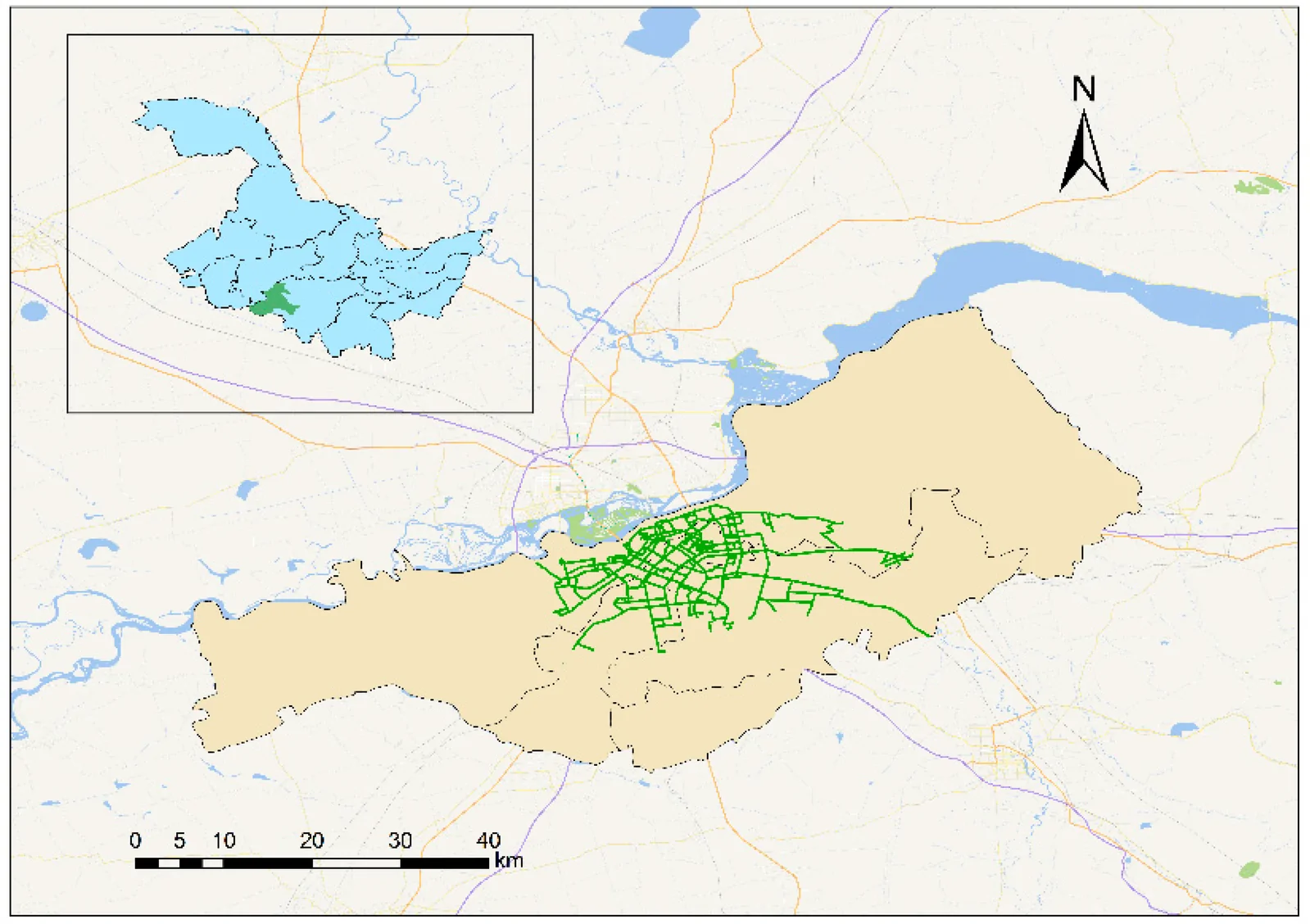
Road network of the Harbin study area.

**Figure 2:**
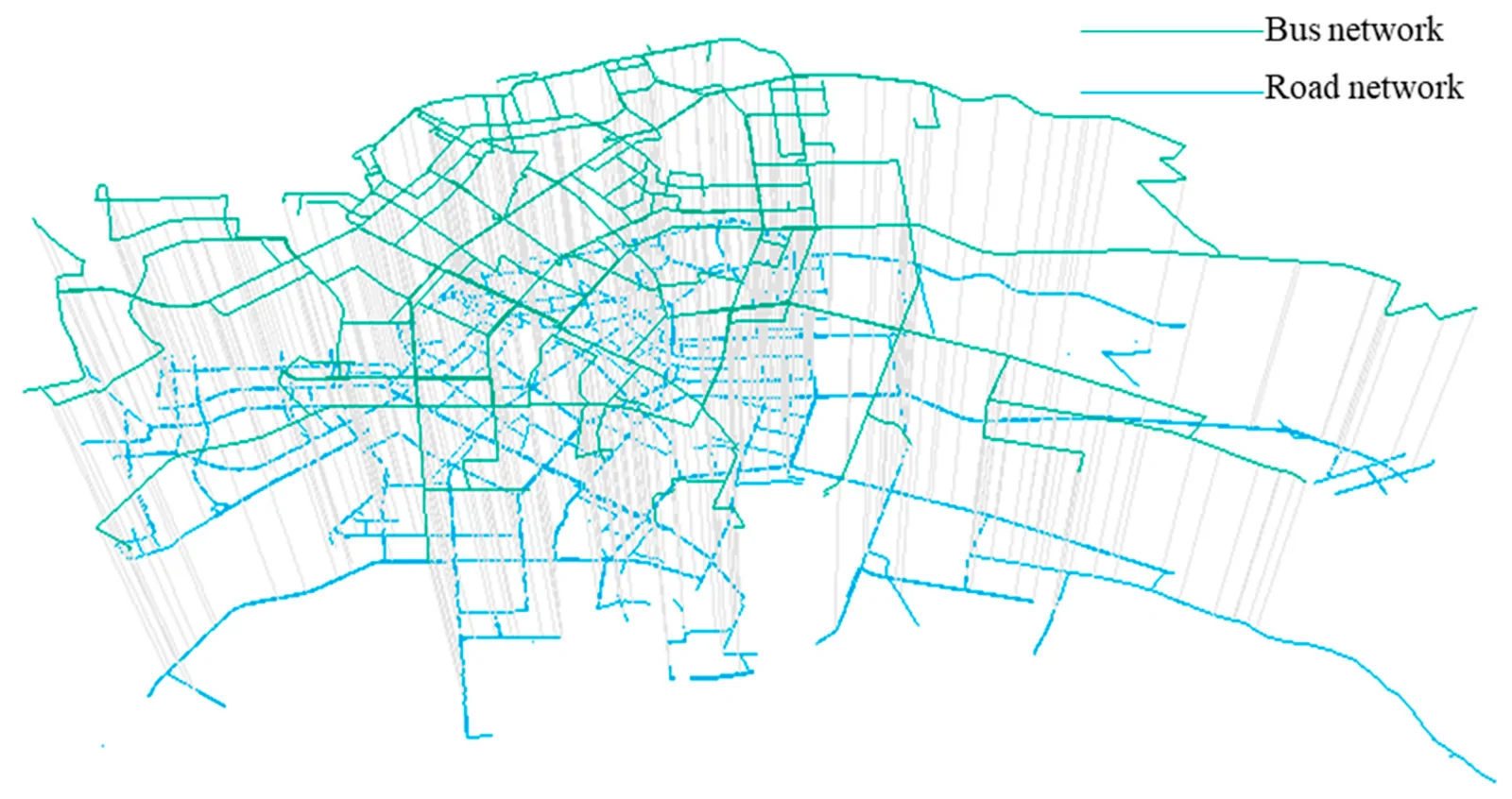
Coupled bus–road network, with the bus network in the upper layer and the road network in the lower layer.

Public transport and road networks coexist both geographically and spatially, with the public transport network relying on the road network to a certain extent. In a real-world bus network, link capacity varies at different times. To capture this dynamic, we constructed an interdependent network combining Harbin’s main bus routes with the corresponding road network. Figure 1 presents the Harbin road network, which serves as the validation example for this study. Figure 2 illustrates the composite road-transit network, with the upper layer representing the bus network and the lower layer depicting the road network.

### 3.3. Destination Estimation

The bus smart-card transaction data used in this study were provided by the Harbin bus company (Harbin, China).

#### 3.3.1. Method of Data with Travel Chain

The daily travel behavior of passengers mainly commuting between their place of residence and their place of work during their travel, and the origin of the last trip of the day is the destination of the first trip of the day. The boarding station of the next trip for a passenger who swiped his card more than once is therefore the boarding station from the previous.

#### 3.3.2. Method of Data Without Travel Chain

In practice, the available IC card data is often incomplete and may not fully capture the entire travel chain. To estimate the destination for the remaining IC card data, we utilize station attraction intensity. The key factors influencing station attraction intensity are passenger travel distance and travel time, considered from both spatial and temporal perspectives. The travel distance can be approximated by the number of bus stops passed, which is modeled using a Poisson distribution. The probability $P_{ij}$ can be expressed:

(5) $$P_{ij}=\left\{\begin{array}{l} \frac{e^{-\lambda }\lambda^{\left(j-i\right)}}{(j-i)!},i<j,\\ 0,i\geq j, \end{array}\right.i,j=1,2,\ldots n$$

where i and j are the serial numbers of the origin and destination stations, respectively. n is the number of stations on the bus line and λ is the mean number of stops in a bus line (based on the survey, Harbin residents can satisfy 10 stations, so in s paper λ = 10). If the number of remaining stops after the boarding stop is fewer than λ, then λ=n−1.

### 3.4. Traffic Assignment Model

As the focus of this paper is on the vulnerability of the bus network considering the real-time traffic dynamics of the road, a traveler chooses the route to her destination. For simplicity, it is assumed that the cost of a road segment for a traveler is equal to the travel time.

#### 3.4.1. Calculation of Marginal Costs

The shortest paths algorithm of Dijkstra is used in this paper to generate path selection sets, and passenger demand is distributed over the network by applying the route choice model. A dynamic passenger assignment is performed by considering the travel cost associated with each route.

The travel cost is composed of the in-vehicle time T and $t_{transfers}$ the number of transfers. The travel cost is conceived in terms of the utility C as follows:

(6) $$C=T+t_{transfers}$$

#### 3.4.2. User-Equilibrium Assignment Model

The user equilibrium model addresses the traffic problem by modeling route choices as follows: passengers complete their journeys by selecting a specific path, denoted as l. The flows that emerge when each passenger minimizes their own travel time are referred to as user equilibrium flows. In this state, it is theoretically impossible for any driver to benefit by deviating from their chosen route. This concept mirrors Wardrop’s first principle of transportation, which describes the Nash equilibrium in road networks: the travel times on all routes used by origin–destination (OD) pairs are equal and are less than or equal to the travel times on any unused route. In essence, the flow distribution in a user equilibrium network reflects the optimal distribution of traffic. The convex program for solving the user equilibrium problem is formulated as follows:

(7) $$\begin{array}{l} \min\;\sum_{l\in L}\int_{0}^{x_{l}}t_{l}\left(x\right)dx\\ s.t.\;\sum_{k}f_{k}^{st}=f^{st}\;\\ \;\;\;\;\;\;\;\;f_{k}^{st}\geq 0,\forall k\\ \;\;\;\;\;\;\;\;x_{l}=\sum_{s}\sum_{t}\sum_{k}f_{k}^{st}\delta_{l,k}^{st} \end{array}$$

where L is the set of all link l in a traffic network, $t_{l}\left(x_{l}\right)$ is the link characteristic function that describes the relationship between the travel time $t_{l}\left(x_{l}\right)$ and the passenger flow $x_{l}$ on link l. The user-equilibrium assignment problem in Equation (7) was solved using the Frank–Wolfe algorithm implemented in TransCAD. Link travel times were calculated using the BPR function in Equation (8). The stopping conditions were set to a gap tolerance of 0.001 and a maximum of 50 iterations, with the procedure terminating when either condition was reached. $f_{k}^{st}$ is the passenger flow between the original node s and the destination node t on path k, $f^{st}$ is total traffic between the original node s and the destination node t, and $\delta_{a,k}^{st}=1$ when section l is on path k. BPR function as follows:

(8) $$t_{l}\left(x_{l}\right)=t_{l}^{0}\left(1+\alpha {\left(\frac{x_{l}}{c_{l}}\right)}^{\beta }\right)$$

where $c_{l}$ is the capacity of a bus segment l, $t_{l}^{0}$ and is the free-flow travel time. The α and β are the shape coefficients, the values of which are set as α = 0.15 and β = 4.0 generally. Since a simple topological structure ignores the heterogeneity of traffic flows in a traffic network, edge weights are introduced to quantify the distribution of traffic flows through travel times $t_{l}\left(x_{l}\right)$. The BPR parameters were set to α = 0.15 and β = 4.0. These values were adopted as modeling assumptions and were not calibrated using Harbin-specific observations.

The UE model is used to determine the initial flow distribution for each traffic scenario before disruption. During the subsequent cascading-failure simulation, flows are redistributed according to the edge-betweenness-based rule in Equations (12) and (13), without re-solving the UE problem after each node failure. The framework therefore combines an initial equilibrium assignment with a simplified post-failure redistribution process and does not represent dynamic user equilibrium throughout the disruption.

### 3.5. Considering the Capacity Reduction of Bus Network

In this subsection, we propose a model to assess link capacity reductions in urban bus networks (UBNs) based on link-specific disruption scenarios. As a simplifying assumption, the effective capacity of each bus link is assumed to decrease in proportion to its operating speed relative to the reference condition. This approximation is used to represent congestion-related service degradation and is not intended as a general physical relationship between speed and capacity. The actual road travel speed is derived from the data presented in the previous section. The steps for this process are as follows:

Step 1: Calculate the origin–destination (OD) matrix of the bus network using the OD estimation model from Section 3.1, based on IC card data.

Step 2: Use the spatial linkage tool in ArcGIS 9.2 to connect the attributes of the road network to the bus network.

Step 3: Retrieve road traffic data for 8:00, 12:00, 17:00, and 19:00 from Google Maps. Then, use the output from Step 2 to determine the actual bus operating speed.

**Figure 3:**
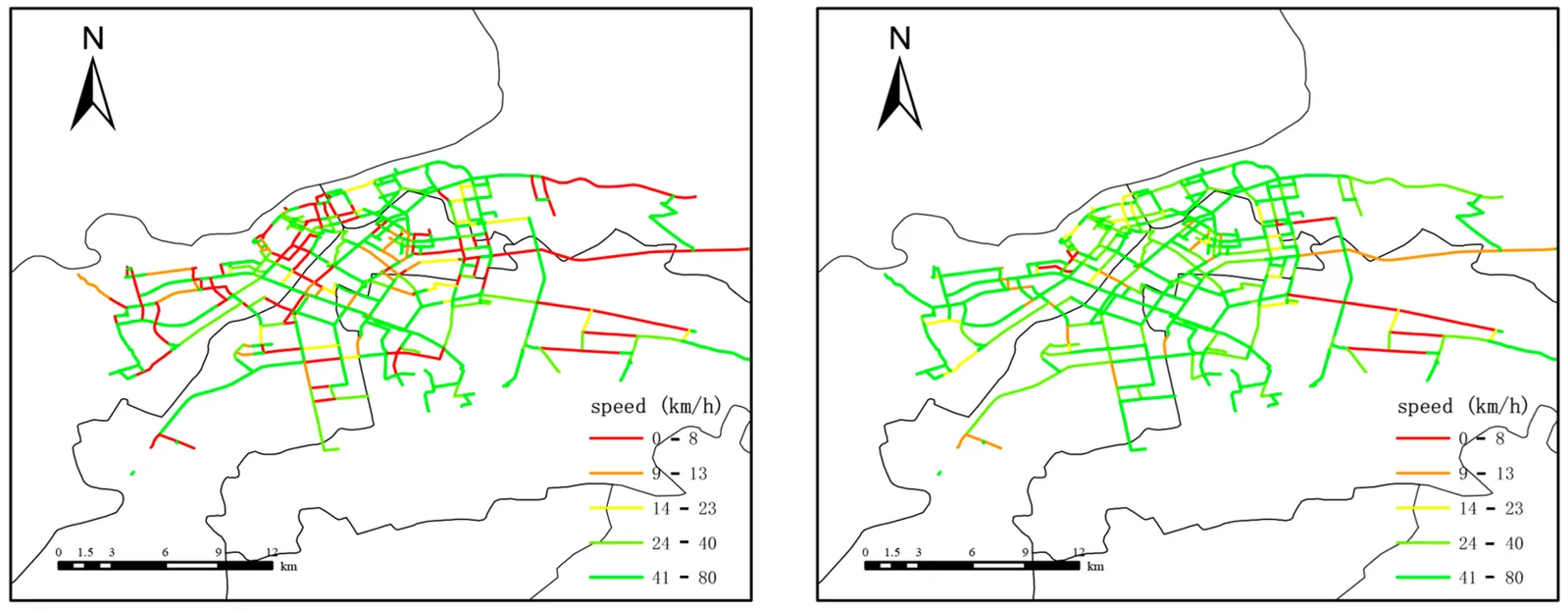
The visualization of the traffic situation of a road network (8:00, 12:00).

**Figure 4:**
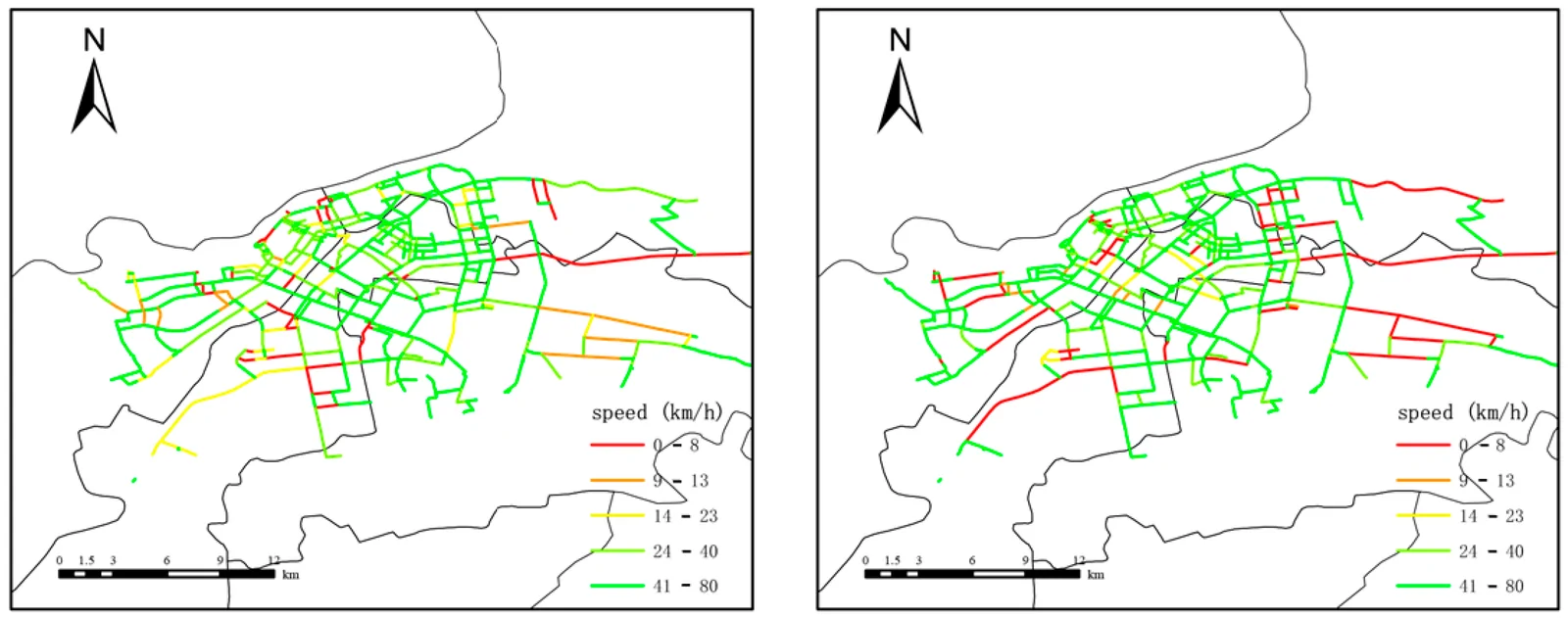
The visualization of the traffic situation of a road network (17:00, 19:00).

Figure 3 and Figure 4 illustrate the road traffic conditions obtained from Steps 1 and 2. The colors in the diagrams represent vehicle speeds, with red indicating congestion and green indicating clear traffic. The graphs clearly show that traffic congestion is most severe at 8:00 AM and 7:00 PM, although the specific segments experiencing congestion vary. Table 1 presents the actual travel times for public transport along with the corresponding reductions in route capacity. While congestion is more prevalent at 7:00 PM compared to 12:00 PM, the shorter average travel time can be attributed to the differing lengths of congestion across various roads. Consequently, this paper analyzes the vulnerability of bus networks under four distinct traffic scenarios.

## 4. Vulnerability Research Model Based on CML

In this subsection, passenger saturation is used as the initial state of the station. And the improved model of CML is proposed based on the topological and passenger flow properties of the nodes. The passenger flow redistribution model is introduced and analyses the cascading-failure vulnerability of public transport networks.

### 4.1. Station State and Failure Model

#### 4.1.1. Initial Node State

The capacity of a bus station depends on the number of bus lines, the frequency of its departures and the passenger load factor in UBNs. Based on this, this paper defines the actual capacity of bus stations, as follows:

(9) $$C_{i}=n_{i}*df_{l}*p_{l}^{busload}$$

where $C_{i}$ denotes the capacity of node i, $n_{i}$ is the number of lines across node i, $df_{l}$ is the departure frequency of the line and $p_{l}^{busload}$ is the busload of the line l.

#### 4.1.2. Improved CML Model for Cascading Failures

The term “improved” refers to the adaptation of the weighted CML framework to bus-network vulnerability analysis under different road traffic conditions. The framework links traffic-dependent bus-link capacity assumptions and passenger-flow assignment to the network inputs used in the cascading-failure analysis. It retains the basic weighted CML structure, while the extension concerns the construction and integration of traffic-scenario-specific inputs rather than a fundamentally new local dynamical map.

In UBNs, node state is not only related to the structural characteristics, but also related to the passenger flow distribution, and the structural characteristics include node degree and edge betweenness, and the passenger flow distribution covers weighted edge betweenness and node strength. In this paper, the node degree, weighted edge betweenness and passenger flow are adopted to design the CML model, and the node state model can be integrated by Formula (10).

(10) $$x_{i}\left(t\right)=\left|\begin{array}{l} \left(1-\tau_{1}-\tau_{2}-\tau_{3}\right)f\left(x_{i}\left(t-1\right)\right)+\tau_{1}\sum_{i}^{j}\frac{e_{ij}f\left(x_{j}\left(t-1\right)\right)}{k_{i}}+\tau_{2}\sum_{i}^{j}\frac{w_{ij}B_{ij}f\left(x_{j}\left(t-1\right)\right)}{TFB_{i}}\\ +\tau_{3}\sum_{i}^{j}\frac{w_{ij}f\left(x_{j}\left(t-1\right)\right)}{S_{i}} \end{array}\right|$$

where $x_{i}\left(t\right)$ is the state of the node $v_{i}$ at time t, which also describes the traffic condition of station $v_{i}$. Meanwhile, $\tau_{1}$, $\tau_{2}$, $\tau_{3}$ are the coupling coefficients of the node degree, traffic flow betweenness, node strength respectively, and $\tau_{1},\tau_{2},\tau_{3}\in \left(0,1\right)$, $\left(\tau_{1}+\tau_{2}+\tau_{3}\right)\leq 1$. The function f demonstrates the local dynamic behaviors which are chosen in this work as the chaotic logistic map, $f\left(x\right)=4x\left(1-x\right)$, to demonstrate the evolution law of volume or capacity constraints of stations.

The CML model integrates the node degree, traffic flow betweenness, and the node strength to describe the node state in Formula (9), and the integration of these aspects is more suitable and comprehensive. These three state factors can reflect the node state in more detail and are likely to correspond to the actual state of the node. Meanwhile, the external perturbation R (R>1) is involved in the expression of the node state to imitate the failure intensity, therefore the Formula (11).

(11) $$x_{i}\left(t\right)=R+\left|\begin{array}{l} \left(1-\tau_{1}-\tau_{2}-\tau_{3}\right)f\left(x_{i}\left(t-1\right)\right)+\tau_{1}\sum_{i}^{j}\frac{e_{ij}f\left(x_{j}\left(t-1\right)\right)}{k_{i}}+\tau_{2}\sum_{i}^{j}\frac{w_{ij}B_{ij}f\left(x_{j}\left(t-1\right)\right)}{TFB_{i}}\\ +\tau_{3}\sum_{i}^{j}\frac{w_{ij}f\left(x_{j}\left(t-1\right)\right)}{S_{i}} \end{array}\right|$$

where R denotes the external perturbation intensity. Node failure is determined by the updated CML state: node *i* fails when *x_i_*(t) > 1. The failed node and its incident edges are then removed, and the resulting flow redistribution affects subsequent node-state updates. The node-state threshold of 1 is distinct from a network-level critical value of *R* or the coupling coefficients. No network-level critical value based on a prescribed failed-node fraction is estimated in this study.

### 4.2. Flow Redistribution Model

When a station fails according to the CML node-state criterion, it becomes unavailable, and its incident edges are removed from the active bus network. The affected passenger flows are then redistributed using Equations (12) and (13), influencing subsequent node-state updates and potentially triggering further failures.

Edge betweenness measures the participation of an edge in shortest paths and is adopted here as a topological proxy for its relative role in accommodating redistributed flows. Accordingly, the affected flows are allocated among the eligible receiving edges in proportion to their betweenness. This simplifying assumption provides a computationally tractable way to represent successive flow transfers during cascading failures. It does not constitute a behavioral route-choice model or guarantee that a new user equilibrium is reached after each failure.

The node connected to $v_{j}$ is defined as $v_{k}$, and the increase in traffic flow on edge $v_{j}$ to $v_{k}$ is defined as follows:

(12) $$\Delta F_{j\to k}=\Delta F_{i\to j}\frac{B_{j\to k}}{\sum_{k=1}^{k\in Neighbor_{j}}B_{j\to k}}$$

where $B_{j\to k}$ is the betweenness of the edge of $e_{jk}$, $\Delta F_{j\to k}$ is the traffic flow of increase for edge of $e_{jk}$; then at the next moment of the failure, the traffic flow of $e_{jk}$ is as follows:

(13) $$F_{j\to k}(t+\Delta t)=F_{j\to k}(t)+\Delta F_{j\to k}=F_{j\to k}(t)+\Delta F_{i\to j}\frac{B_{j\to k}}{\sum_{k=1}^{k\in Neighbor_{j}}B_{j\to k}}$$

Here, the updated flow represents the flow on the corresponding edge after redistribution. Flow redistribution affects subsequent node-state updates through the CML framework. A node fails when its updated state exceeds 1, after which its incident edges are removed from the active network. No independent edge-removal criterion based on a flow-capacity threshold $F_{jk,max}$ is imposed.

### 4.3. Vulnerability Metric

In this subsection, the critical stations are selected to suffer attacks with different evaluation metrics to analyze the vulnerability of UBNs. The station and the edges connected with this station are removed from the UBNs in case of station failure. The cascading-failure ratio was defined for the entire network to assess the vulnerability of UBNs based on these attack strategies, as follows:

(14) M=NremaintNtotal

where $N_{remain}^{t}$ is the number of nodes after cascading failures and $N_{total}$ the total number of nodes at graph G.

## 5. Research Results

Transportation networks can be disrupted by natural disasters, traffic accidents, and incidents related to public safety. Events such as earthquakes, hurricanes, floods, and other emergencies severely impact urban transportation systems. Traffic accidents, power outages, and other incidents can lead to large-scale congestion. In this study, network disruptions are simulated through deliberate attacks, where nodes are ranked by importance and targeted sequentially.

The vulnerability of urban bus networks (UBNs) is assessed using a Coupled Map Lattice (CML) model, which explores cascading failures by analyzing passenger flow dynamics. The primary contribution of this method is its ability to not only capture the risks associated with high passenger flow during cascading failures but also quantify the system’s resilience by considering topological redundancy and the capacity of different stations.

This section examines the cascade failure vulnerability of the urban public transport network under varying traffic dynamics, following the failure of key stations. The analysis is based on node importance, the cascading-failure model, and the vulnerability model outlined in Section 4 and Section 5.

### 5.1. Vulnerability of Different Attacks

Four attack strategies were considered: largest-degree attack (LDA), high traffic-flow-betweenness attack (HTA), largest-strength attack (LSA), and random attack. The first three are targeted attack strategies. For each scenario, the random-attack results were averaged over 50 simulations. Individual-run outputs were not retained; therefore, standard deviations could not be calculated, and run-to-run variability remains unquantified.

**Figure 5:**
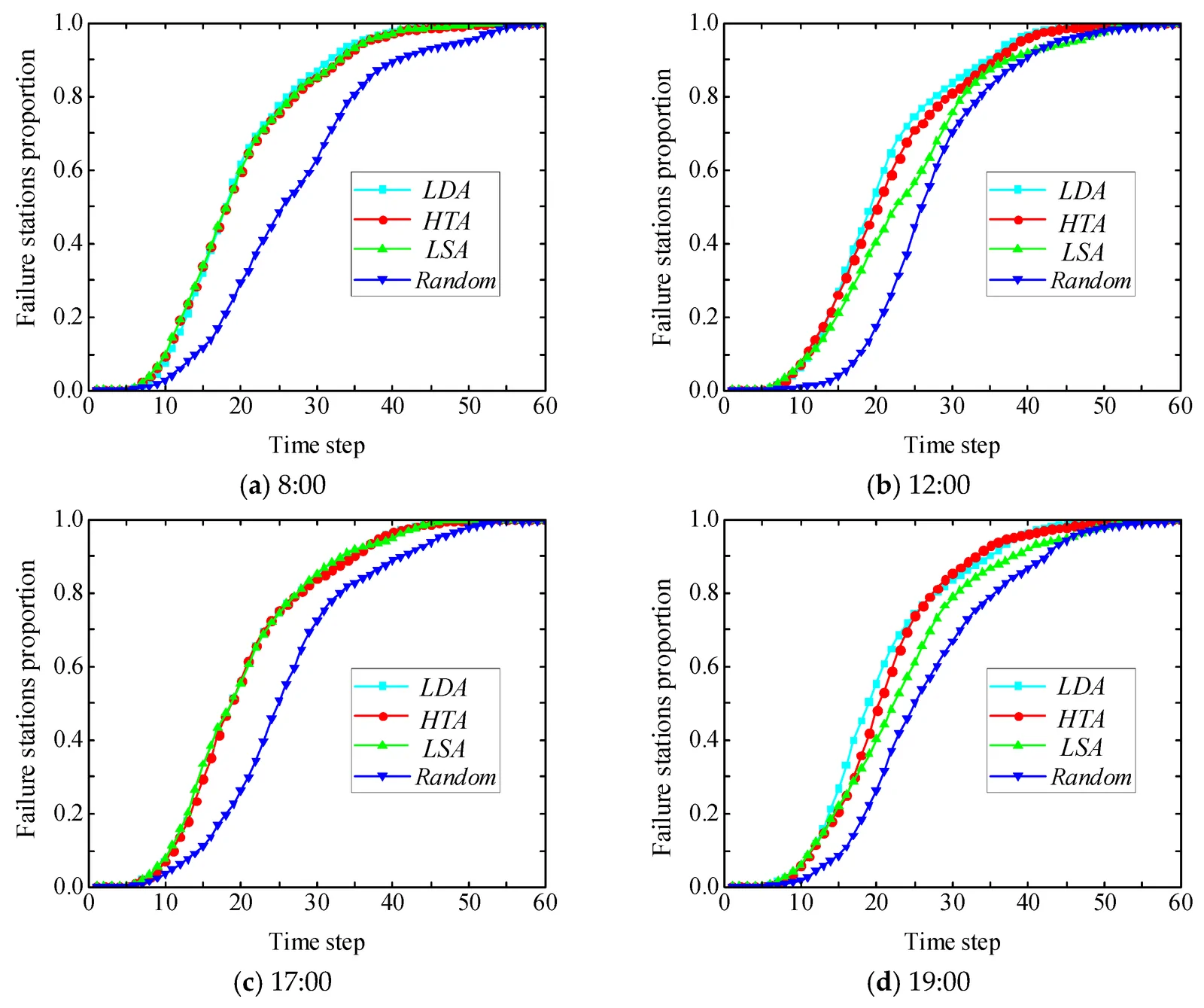
Vulnerability of the four attack modes with time under different traffic situation.

As we can see from Figure 5, various deliberate attacks can result in different vulnerabilities and the four moments also exhibit different vulnerability characteristics in the weighted UBNs. Approximately normally distributed is the relationship between cascading-failures and time steps, in which the speed of failure increases from fast to slow. Moreover, different critical stations with failures have different peak time steps and convergence time steps for cascading failures. However, the LDA has the strongest vulnerability under the four different moments. The cascading failure speed peaks of UBNs occur earlier when road conditions are weak (8:00, 19:00). The poor condition of roads is evident earlier than the good condition of roads about the peak time step of cascade failure. Consequently, public transportation networks should have different response times for operation management and emergency response plans at different times.

### 5.2. Vulnerability of the Different Perturbation Values

This section examines the effects of external perturbation intensity *R* on cascading failures under different traffic conditions and three targeted attack strategies: LDA, HTA, and LSA. The coupling coefficient is fixed at 0.3, and four perturbation values, *R* = 1.5, 1.8, 2.1, and 2.4, are tested. These values are selected for scenario comparison rather than for identifying critical perturbation thresholds.

**Figure 6:**
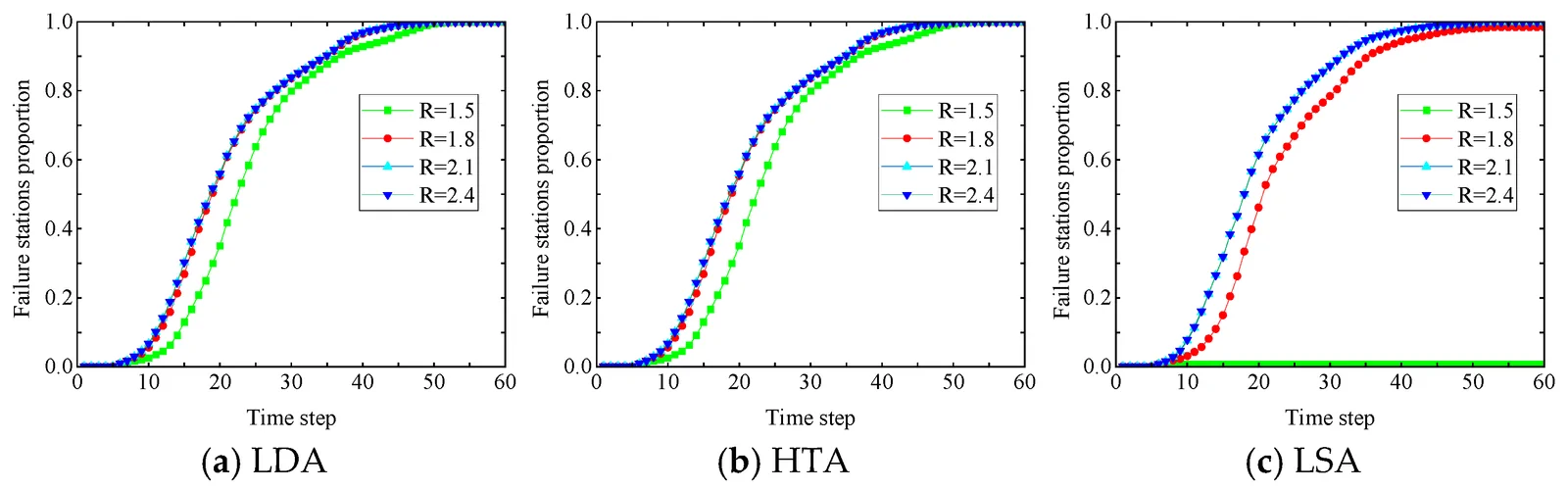
Network vulnerability under three deliberate attacks with different R when 8:00.

**Figure 7:**
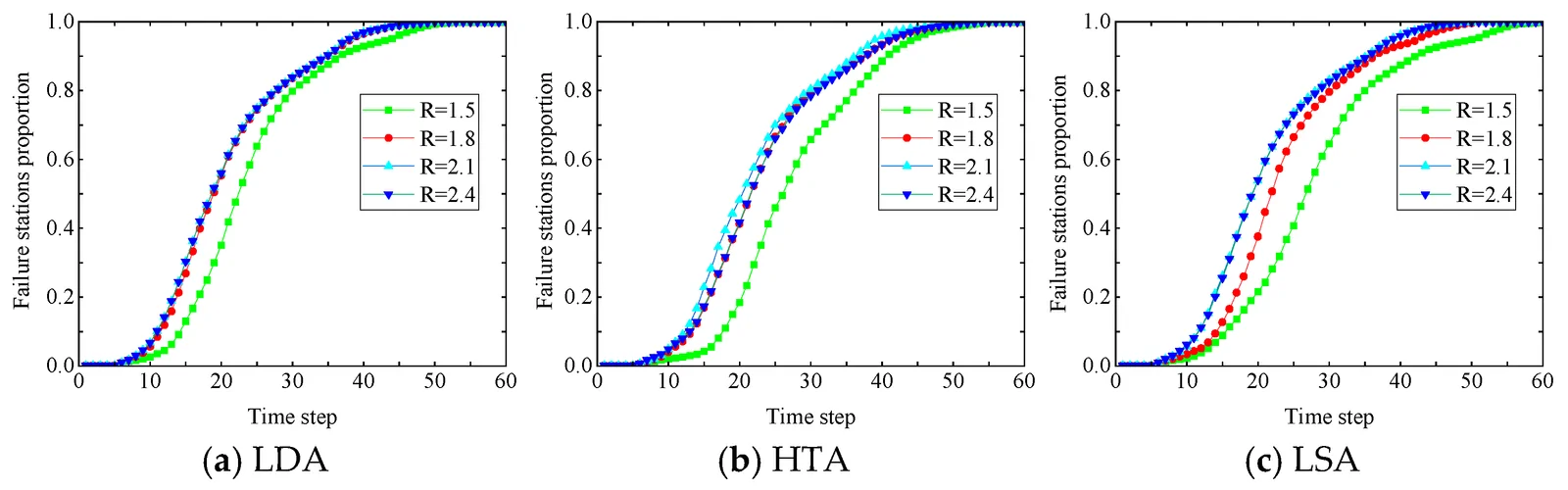
Network vulnerability under three deliberate attacks with different R when 12:00.

**Figure 8:**
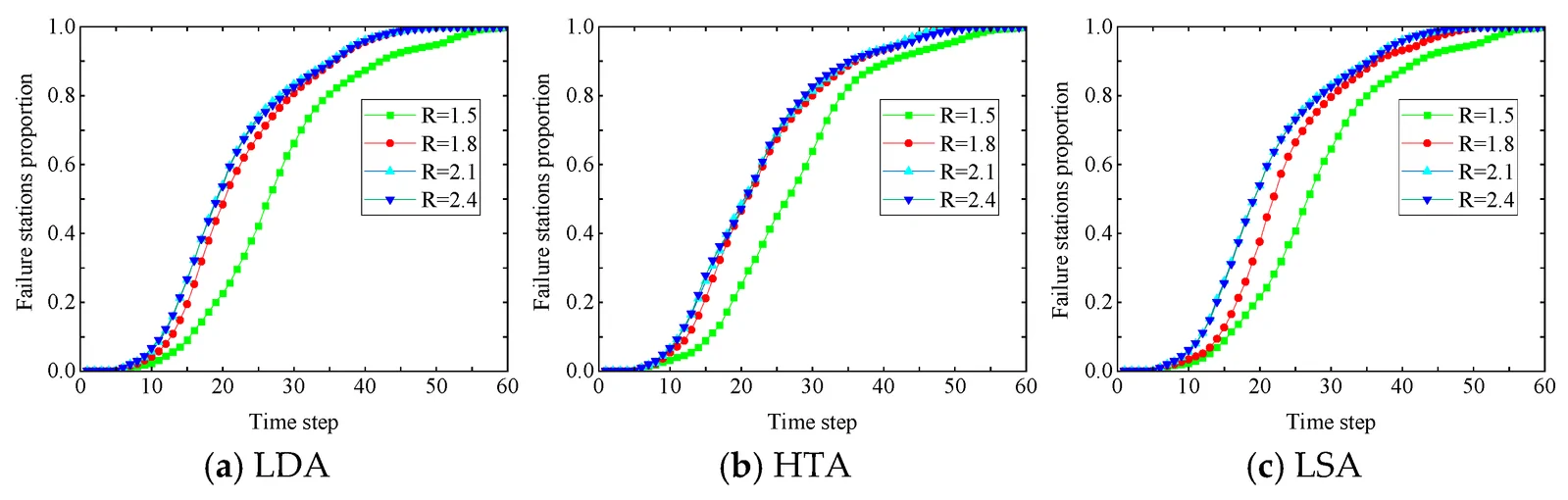
Network vulnerability under three deliberate attacks with different R when 17:00.

**Figure 9:**
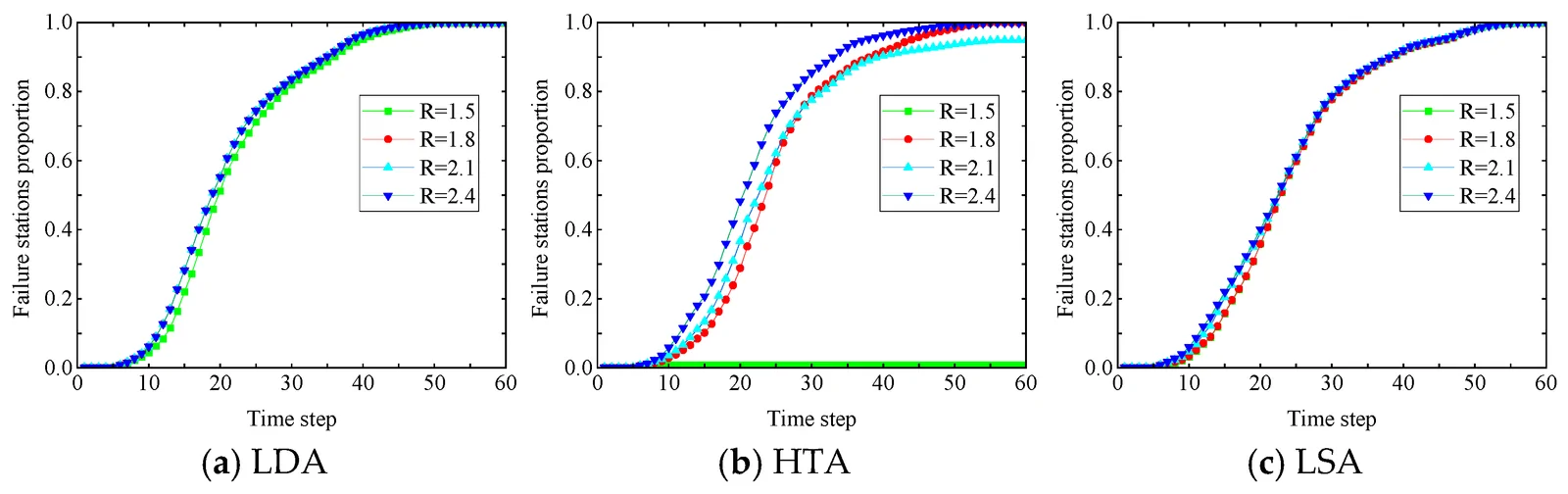
Network vulnerability under three deliberate attacks with different R when 19:00.

Figure 6, Figure 7, Figure 8 and Figure 9 compare cascading-failure responses across the tested perturbation values. Increasing R generally accelerates failure propagation, although the responses vary with attack strategy and traffic condition. The tested perturbation values of 1.5 and 1.8 yield similar peak propagation timing in several scenarios. At the higher tested values of R = 2.1 and R = 2.1, the response curves become largely similar, suggesting saturation of the simulated response over this sampled range under the tested settings. Differences among LDA, HTA, and LSA reflect their scenario-dependent responses to perturbation intensity rather than distinct, precisely determined critical values. These observations describe the results at the sampled parameter settings and do not establish a lower or upper perturbation threshold.

### 5.3. Vulnerability of the Different Couple Strengths

Coupling strengths between stations are a measure of their degree of correlation. The closer the interaction between the stations, the faster the fault propagation with high τ. Therefore, it is necessary to study the effect of different variables on the cascade failure of UBNs.

**Figure 10:**
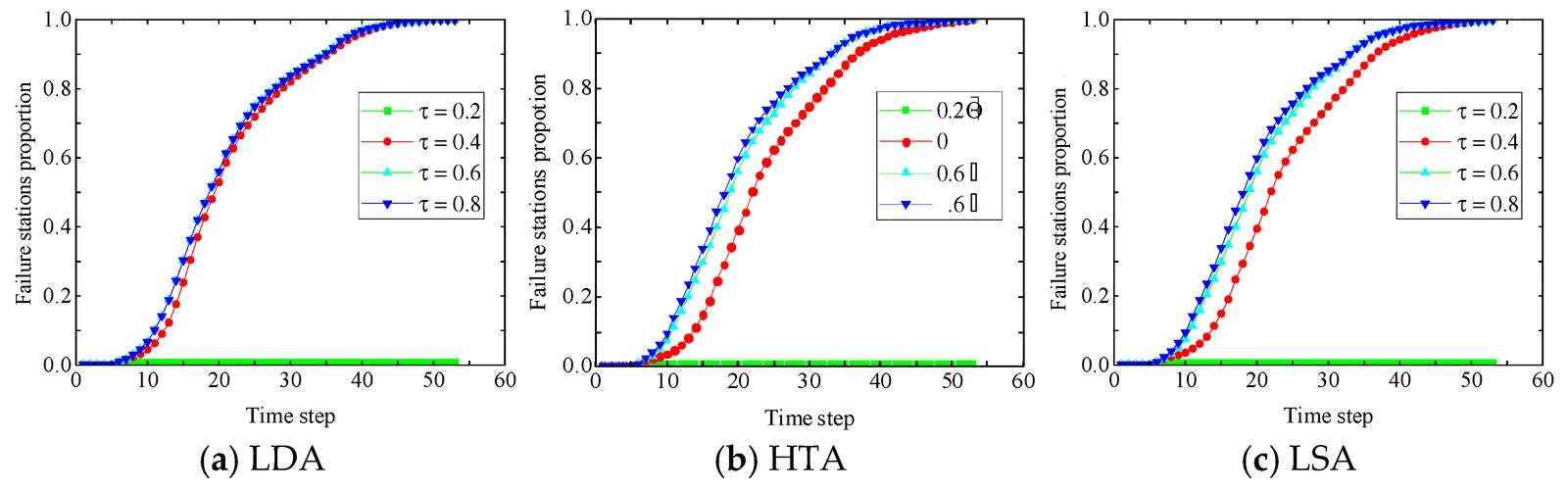
Network vulnerability under three deliberate attacks effect of coupling coefficient τ when 8:00.

**Figure 11:**
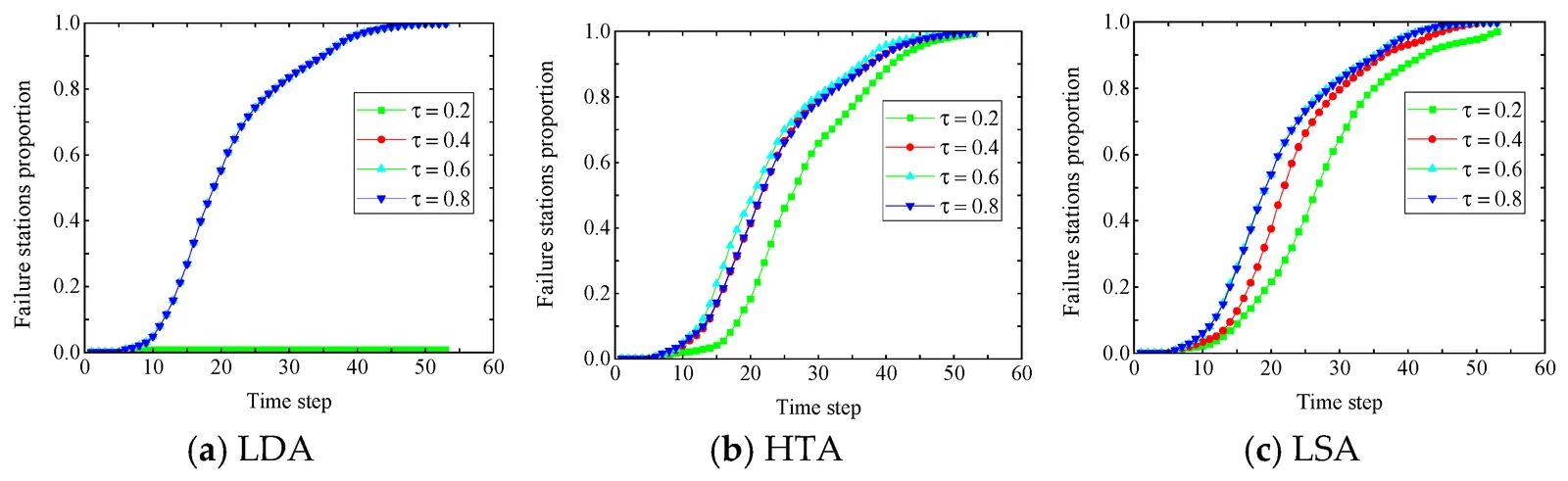
Network vulnerability under three deliberate attacks effect of coupling coefficient τ when 12:00.

**Figure 12:**
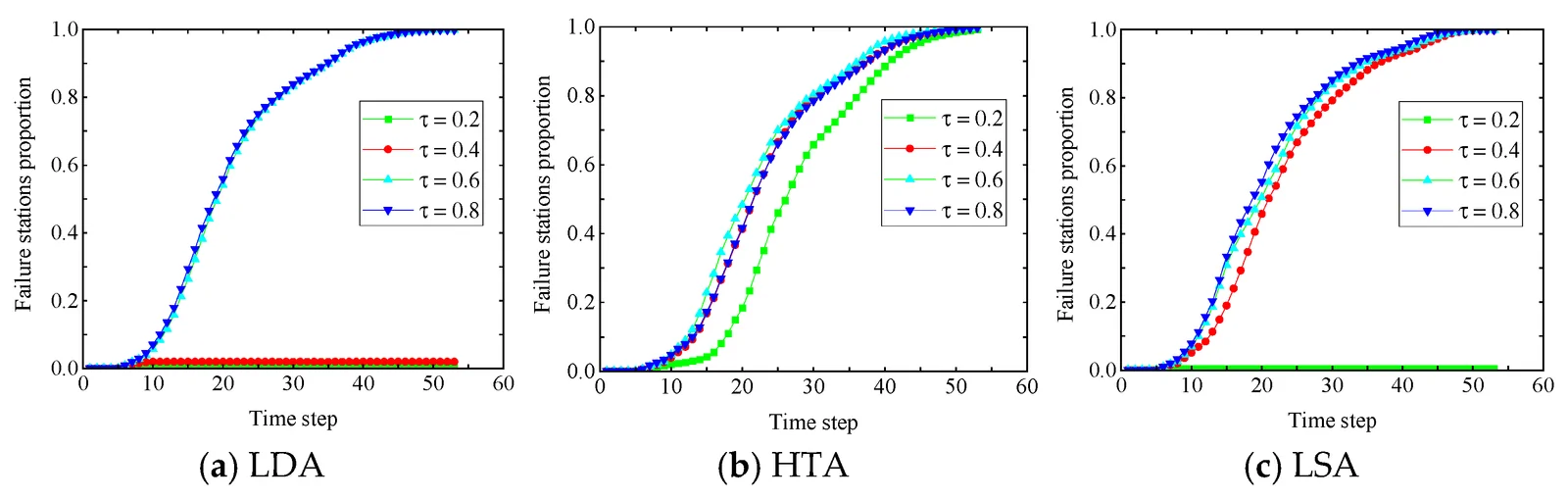
Network vulnerability under three deliberate attacks effect of coupling coefficient τ when 17:00.

**Figure 13:**
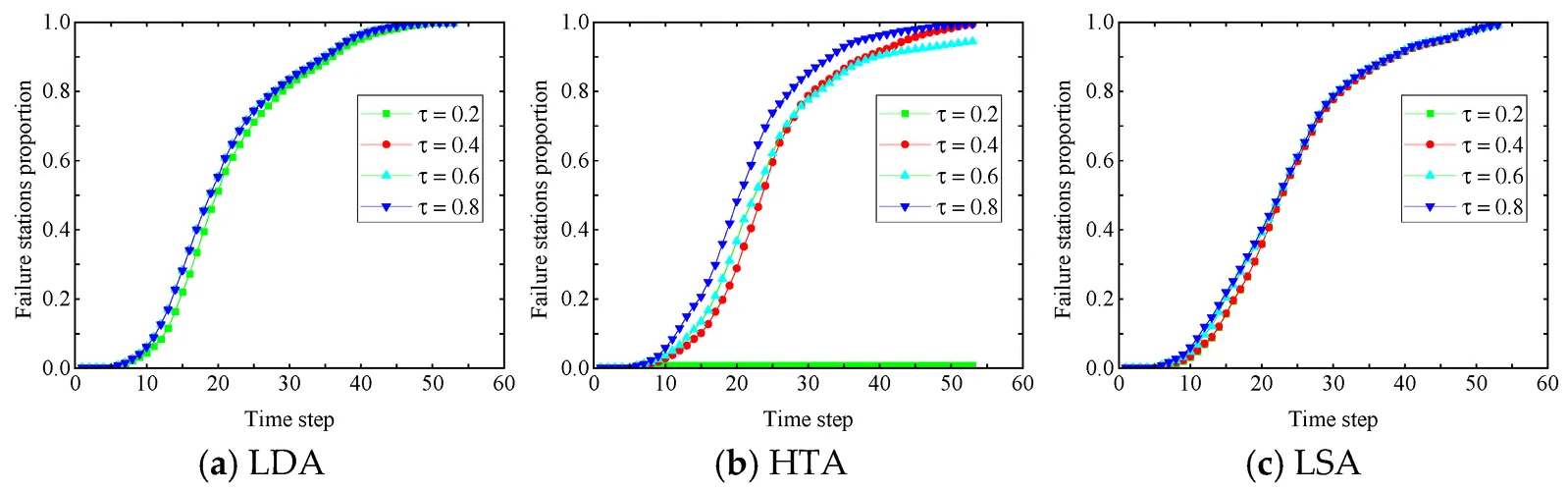
Network vulnerability under three deliberate attacks effect of coupling coefficient τ when 19:00.

Figure 10, Figure 11, Figure 12 and Figure 13 show that the influence of coupling strength depends on the attack strategy and traffic conditions. Some curves overlap closely, whereas others exhibit differences in the timing and extent of failure propagation. The values 0.2, 0.4, 0.6, and 0.8 are the tested coupling settings and are not reported as precisely determined network-level failure thresholds. These comparisons characterize the model response at the sampled parameter values; a critical coupling value would require an explicit network-level failure criterion and a more detailed parameter search.

## 6. Conclusions

This study constructs a weighted urban bus networks under different traffic conditions using complex network theory and empirical public transport data. An improved CML framework incorporating passenger-flow redistribution is used to assess network vulnerability and examine cascading-failure dynamics.

The results show that cascading-failure responses vary across attack strategies and traffic scenarios. Within the tested parameter ranges, changes in external perturbation intensity and coupling strength affect the timing and extent of failure propagation, although the responses differ across scenarios. These findings are conditional on the model assumptions and parameter settings. The tested values of *R* and the coupling coefficients should not be interpreted as universal or precisely determined network-level failure thresholds.

From a practical perspective, differences in peak propagation timing across traffic conditions can help bus operators and traffic management authorities identify periods requiring earlier monitoring and emergency response. Stations identified as vulnerable under particular traffic scenarios can be prioritized for protective measures, passenger guidance, and contingency resource allocation. The simulated sensitivity to disturbances can also support comparisons of time-specific passenger-flow management strategies, while specific interventions require validation against local observations and operational constraints.

This study has several limitations. The proportional capacity–speed assumption does not explicitly account for service headways, vehicle capacities, dwell times, or operational adjustments, which may affect the simulated cascading-failure responses. The betweenness-based redistribution rule also simplifies post-disruption passenger responses and does not capture the re-establishment of user equilibrium. The analysis is also limited to the Harbin trunk bus network and four discrete traffic snapshots, restricting its generalizability and representation of continuous traffic evolution. The results should therefore be interpreted as conditional scenario outcomes rather than directly applicable operational criteria. Future work should calibrate the capacity model, assess sensitivity to the capacity and redistribution assumptions, explore repeated equilibrium assignment or dynamic traffic assignment during disruptions, and extend the analysis to multiple cities, higher-resolution temporal traffic data, and a broader range of disruption scenarios.

## Figures and Tables

**Table 1:** Average travel time and proportion of link capacity.

Time	Average Travel Time (h)	Link Capacity Reduction (%)
8:00	2015.2	34.8
12:00	2270.3	21.2
17:00	1782.8	11.8
19:00	2045.2	23.6

## Data Availability

The original contributions presented in this study are included in the article. Further inquiries can be directed to the corresponding authors.
